# Marine Resources Offer New Compounds and Strategies for the Treatment of Skin and Soft Tissue Infections

**DOI:** 10.3390/md21070387

**Published:** 2023-06-29

**Authors:** Antje Labes

**Affiliations:** Department of Energy and Biotechnology, Flensburg University of Applied Sciences ZAiT, Kanzleistraße 91-93, D-24943 Flensburg, Germany; antje.labes@hs-flensburg.de

**Keywords:** secondary products, sustainable use of the sea, marine biotechnology, bottlenecks, antibiotic resistance

## Abstract

Bioprospecting of the marine environment for drug development has gained much attention in recent years owing to its massive chemical and biological diversity. Drugs for the treatment of skin and soft tissue infections have become part of the search, mainly with respect to enlarging the number of available antibiotics, with a special focus on multidrug-resistant Gram-positive bacteria, being the major causative agents in this field. Marine resources offer novel natural products with distinct biological activities of pharmaceutical importance, having the chance to provide new chemical scaffolds and new modes of action. New studies advance the field by proposing new strategies derived from an ecosystemic understanding for preventive activities against biofilms and new compounds suitable as disinfectants, which sustain the natural flora of the skin. Still, the development of new compounds is often stuck at the discovery level, as marine biotechnology also needs to overcome technological bottlenecks in drug development. This review summarizes its potential and shows these bottlenecks and new approaches.

## 1. Introduction: Skin and Soft Tissue Infections

Skin and soft tissue infections (SSTIs) are a group of infectious diseases that affect the skin and subcutaneous tissue. They are a common cause of morbidity and mortality worldwide, especially in developing countries. SSTIs can occur in any part of the body and are caused by a wide range of microorganisms, including bacteria, fungi, and viruses [1].

The skin is the largest organ in the body and acts as a barrier against environmental insults, including microbial invasion. The skin contains many resident microorganisms that can provide protection against pathogenic microorganisms. However, when the skin barrier is compromised, these resident microorganisms can cause infections, as most of them are opportunistic pathogens [2]. According to estimates, representatives of the *Cutibacterium*, *Staphylococcus*, and *Corynebacterium* genera, isolated from almost all skin areas, may constitute 45 to 80% of the entire skin microbiome [2]. This ambivalent nature of the human skin flora is an important issue in the prevention and treatment of SSTIs. Even more, many mammals share certain groups of skin bacteria, including pathogens, paving the path to zoonoses [3]. In light of increasing antibiotic resistance, such intra-species infections need tight control.

Soft tissue refers to the connective tissue that supports and connects other tissues and organs in the body. It includes muscles, tendons, ligaments, fascia, and adipose tissue. Soft tissue infections can occur when microorganisms invade and multiply in these tissues, leading to inflammation, and they might cause sepsis after generalization from the local infection. Soft tissue infection occurs often after injuries with the involvement of skin bacteria as well as gut-associated microbes.

SSTIs encompass a wide spectrum of clinical presentations, ranging from simple cellulitis to rapidly progressive necrotizing fasciitis. Mild SSTIs are usually self-limited and can be treated with topical or oral antimicrobial agents. Severe SSTIs can be life threatening and require urgent surgical and medical intervention. Acute bacterial skin and soft tissue infections are among the most common reasons for the hospitalization of adults today. Multidrug resistance in the causative pathogens may convert previously mild infections into a life-threatening situation [4]. Diagnosing the exact extent of the disease is critical for the successful management of a patient with a soft-tissue infection.

## 2. Causative Agents

SSTIs are caused by a wide range of pathogens. For all pathogens, the risk of generalization of the local infection to sepsis can be observed. Multidrug resistance is an issue for all groups. The most common causative pathogens of SSTIs are bacteria, including *Staphylococcus aureus*, *Streptococcus pyogenes*, and *Pseudomonas aeruginosa* [5]. These bacteria can cause a range of infections, from mild, superficial infections to severe, life-threatening infections. Staphylococci and Streptococci are part of the human residential flora, but also occur in other animals and can be spread via food [6,7]. 

*Staphylococcus aureus* is a Gram-positive bacterium causing a range of infections, from mild impetigo and folliculitis to severe infections such as cellulitis and necrotizing fasciitis. Methicillin-resistant *S. aureus* (MRSA) has emerged as a significant cause of SSTIs, especially in hospital settings. MRSA is resistant to many antibiotics, making it difficult to treat, and can cause severe and life-threatening infections [6].

*Streptococcus pyogenes*, also known as group A Streptococcus, is a Gram-positive bacterium that is a common cause of SSTIs, especially in children. It can cause a range of infections, including impetigo, erysipelas, and cellulitis. *S. pyogenes* can also cause more severe infections, such as necrotizing fasciitis and streptococcal toxic shock syndrome [7].

*Pseudomonas aeruginosa* is a Gram-negative bacterium that is common, especially, in hospital settings. It can cause a range of infections, including wound infections, burn infections, and folliculitis. *P. aeruginosa* is also a significant cause of healthcare-associated infections, especially in patients with compromised immune systems [8]. Complicated SSTIs caused by resistant Gram-negative bacteria such as *P. aeruginosa* are increasing particularly in vulnerable patients with long-standing infections, those in long-term care facilities, and those with a history of recent hospitalization or prior antibiotic therapy. Mixed infections also occur in up to 25% of SSTIs, and inappropriate therapy was reported in 40% of cases [4].

In addition to bacteria, fungi can also cause SSTIs. The most common fungal pathogens causing SSTIs are *Candida* spp. and *Aspergillus* spp. The normal colonization of human skin with fungal communities was doubted, even though it had been observed for decades. Nowadays, different fungi including *Malassezia*, *Cryptococcus*, *Rhodotorula*, and *Candida* species have been identified as human skin commensals [9,10]. *Candida* spp. can cause a range of infections, from superficial infections such as diaper rash and thrush to severe infections such as deep-seated candidiasis. *Aspergillus* spp. are ubiquitous fungi that can cause a range of infections, from allergic bronchopulmonary aspergillosis to invasive aspergillosis. Dermatophytes comprise a number of filamentous fungi that have the capability to invade keratinized tissue (skin, hair, and nails) of humans and other animals to produce an infection, dermatophytosis, commonly referred to as ringworm. Infection is generally cutaneous and restricted to the nonliving cornified layers because of the inability of the fungi to penetrate the deeper tissues or organs of immunocompetent hosts [11].

Viruses can also cause SSTIs, although they are less common than bacterial and fungal infections. The most common viral pathogens causing SSTIs are the herpes simplex virus and the varicella-zoster virus [12]. The human papilloma virus (HPV) is the main causative agent of various wart types such as verruca vulgaris, plantar warts, and condyloma acuminatum as well as a variety of cancers, and several high-risk strains are currently vaccinated against [13].

The risk factors for SSTIs vary depending on the causative pathogen. Risk factors for bacterial SSTIs include diabetes mellitus, immunosuppression, obesity, peripheral vascular disease, trauma, and intravenous drug use. Risk factors for fungal SSTIs include immunosuppression, HIV/AIDS, and the prolonged use of antibiotics or corticosteroids. Risk factors for viral SSTIs include immunosuppression and close contact with infected individuals [14].

## 3. Classical Treatment Approaches

Classical treatment approaches for SSTIs involve a combination of pharmacological interventions and supportive care to effectively manage and resolve these infections. The primary goal is to eliminate the infection-causing pathogens, alleviate symptoms, promote wound healing, and prevent further complications. Treatment of SSTIs depends on the severity, location, and etiology of the infection. Mild SSTIs can be treated with local antiseptics to avoid spreading the infection and with topical or oral antimicrobial agents, while severe SSTIs may require surgical debridement and global intervention [15,16,17].

Empirical antibiotic therapy is often initiated based on the suspected etiology of the infection. In many cases, broad-spectrum antibiotics are the drugs of choice. Commonly prescribed antibiotics for skin and soft tissue infections include penicillinase-resistant penicillins (e.g., dicloxacillin), cephalosporins (e.g., cefazolin), or clindamycin. The choice of antibiotic is guided by factors such as the severity of infection, local antimicrobial resistance patterns, and patient-specific considerations (e.g., allergies). In some cases, combination therapy may be necessary, especially for severe infections or those caused by drug-resistant bacteria [18]. Proper wound care is essential for promoting healing and preventing the spread of infection. This typically involves cleansing the wound with mild antiseptic solutions, such as chlorhexidine or povidone-iodine. Dead tissue and debris may be removed through debridement. In cases of abscess formation, incision and drainage may be performed to evacuate pus and relieve pressure. It is important to ensure adequate drainage and consider the use of wound dressings appropriate for specific wound characteristics [17]. As SSTI can be associated with significant pain and discomfort, nonsteroidal anti-inflammatory drugs (NSAIDs), such as ibuprofen or naproxen, are commonly used to alleviate pain and reduce inflammation [19].

Classical treatment approaches for skin and soft tissue infections can contribute to the development of multidrug-resistant organisms, leading to multiresistance problems in hospitals including cross-resistance; i.e., the development of resistance to the whole group of antibiotics targeting the same pathogen structure. The situation of multidrug resistance in the treatment of skin and soft tissue infections has become a significant global concern [20]. Over the past few decades, there has been a rise in the prevalence of multidrug-resistant organisms causing skin and soft tissue infections. Bacteria such as *Staphylococcus aureus*, including methicillin-resistant Staphylococcus aureus (MRSA), and *Streptococcus pyogenes* are among the common culprits. These organisms have acquired resistance mechanisms through genetic mutations or the acquisition of resistance genes from other bacteria. 

There is strong evidence suggesting that inappropriate antibiotic treatment is given to approximately 20–25% of patients, causing additional threats for multidrug resistance development [21]. Moreover, the inappropriate use of human-targeted antibiotics in veterinary medicine is a strong driver of the rising numbers of multidrug resistance [22]. The consequences of multidrug resistance in skin and soft tissue infections are profound. Treatment options become limited, as many previously effective antibiotics are no longer effective against these resistant organisms. This can lead to treatment failure, prolonged illness, increased healthcare costs, and a higher risk of complications. The situation is further complicated by the ability of multidrug-resistant bacteria to spread within healthcare settings. Close proximity between infected and susceptible patients, inadequate hand hygiene practices, and contaminated medical equipment all contribute to the transmission and dissemination of these resistant organisms. This creates a challenging environment in hospitals, where patients with skin and soft tissue infections may be at increased risk of acquiring multidrug-resistant strains [23].

To address the situation of multidrug resistance in the treatment of skin and soft tissue infections, a comprehensive approach is required, including the development of new antibiotics having novel targets or improved stability against degrading resistance mechanisms. Daptomycin, a microbial lipopeptide, was a successful example of a new mode of action against MRSA, which enabled treatment of patients having complicated SSTI caused by resistant Gram-positive pathogens including vancomycin and linezolid resistances (both are reserve antibiotics) [24]. The development of new antibiotics and alternative treatment modalities is essential, but the search for new antibiotic scaffolds addressing new targets will be an ongoing search as any use of antibiotic compounds will also lead to resistant pathogens. However, new agents displaying new scaffolds and modes of action are desperately needed worldwide—the pipelines are rather empty and will also need continuous refill [25].

## 4. Marine Secondary Metabolites as a Source

Secondary metabolites are chemical compounds produced by microorganisms, plants, and animals that have a number of biological functions, such as defense mechanisms against pathogens. Their ecological role can serve as a blueprint for pharmaceutical application, as secondary metabolites can have a variety of mechanisms of action against pathogens. For example, some compounds can disrupt bacterial cell membranes, while others can interfere with bacterial cell wall synthesis or inhibit bacterial enzymes. Other compounds can interfere with quorum sensing, a bacterial communication system that plays a critical role in virulence and biofilm formation. Since the golden era of antibiotics in the early 20th century, secondary metabolites, especially from microbes were the source or model for new antibiotics [26]. However, the decline in the number of new chemical scaffolds discovered and the rediscovery problem of old known molecules has become a limitation for discovery programs developed by an industry confronted with a lack of incentives and a broken economic model. Advances in genome mining have confirmed the richness of biosynthetic gene clusters (BGCs) in the majority of microbial sources, and this suggests that an untapped chemical diversity is waiting to be discovered [27]. 

A strategy to unravel the biosynthetic potential is to untap so-far underexplored habitats for biodiscovery with the aim of avoiding rediscovery. The world’s ocean seems to have promising biodiversity with ecological features that may favor the production of antimicrobial and anti-biofilm metabolites. Accordingly, screening campaigns revealed marine organisms as a particularly rich source of secondary metabolites with potential antimicrobial activity. Many marine organisms, such as sponges, corals, and algae, produce secondary metabolites as part of their defense against microbial pathogens in their environment necessary for organisms with a sessile lifestyle in a water environment [28,29]. Bioprospecting of the marine environment has gained much attention in recent years owing to its massive chemical and biological diversity [30]. A variety of bioprospecting techniques (including cultivation-dependent to independent approaches) have been described so far toward harnessing the bioactive potential of marine macrobes [31]. Particularly, sessile organisms, such as marine sponges, have been shown to be abundant reservoirs of novel natural products with distinct biological activities of pharmaceutical importance, but, very often, the microbiome of these macroorganisms has been shown to be the true producer of the bioactive compounds, or at least contributing to their biosynthesis. Next to anti-cancer activities, antimicrobial activities have been the focus of biodiscovery campaigns. A wide spectrum of compounds and extracts possessing antibacterial and/or anti-biofilm activities have been reported [32]. A detailed analysis by Kong et al. showed that large portions of marine scaffolds are novel [33].

A class of marine-derived secondary metabolites with potential activity against SSTIs is peptides. Many marine organisms, including sponges and sea anemones, produce peptides that have antimicrobial activity. These peptides have diverse structures and mechanisms of action and have been shown to have activity against a range of bacterial and fungal pathogens [34]. Almost all marine organisms produce natural antibacterial compounds as an essential line of defense to survive. Antimicrobial peptides quite often display broad-spectrum antibiotic activity and may be used as empirical antibiotics. A recent example is anisaxins, membrane-active and antimicrobial peptides from marine parasites, *Anisakis* spp., with potent bactericidal activity and selectivity toward multidrug-resistant Gram-negative bacteria [35]. Nonribosomal peptide synthetases are the major producing enzymes in microbes; genetic screening may be used to identify interesting new structures, which, in the future, may also be produced heterologously [36].

As peptide derivatives, β-lactam antibiotics can be found in the marine environment. Already in the 1950s, cephalosporin C, a β-lactam type natural antibiotic, was discovered from a *Cephalosporium* (nowadays, *Acremonium*) species obtained off the Sardinian coast, being the first fungal antibiotic from a marine environment. In the late 1970s, gliotoxin was identified as a new type of the antibiotic, diketopiperazine, which is produced by an *Aspergillus* sp. strain isolated from marine mud of the Seto Inland Sea. It was the first antimicrobial compound of this type obtained from a fungus originating from deep-sea sediments (for more references on early discoveries of marine fungal antibiotics see [37]). 

Another class of marine-derived secondary metabolites with potential activity against SSTIs is terpenoids. Terpenoids are a large and structurally diverse class of compounds found in many marine organisms, including algae, sponges, and soft corals. Terpenoids have been shown to have antibacterial, antifungal, and antiviral activity, making them promising candidates for the prevention and treatment of SSTIs. Notably, marine fungi and brown algae have been found to be rich sources of terpenoids [38]. Despite a common biosynthetic origin, the final diversity causing enzymatic conversions is quite often rarely studied. The discovery and biochemical characterization of new enzymes would bring to light cryptic natural products, unveil novel cyclization reactions, and allow for more informed bioinformatic predictions. Zhang et al. recently described the discovery and in vivo characterization of a cryptic bifunctional type I diterpene synthase from a marine-derived fungus that synthesizes a tricyclic 5–8–6 hydrocarbon skeleton. The resulting talaronids provide a backbone for anti-streptococcal compounds [39]. 

Polyketides are a large class of compounds produced by marine organisms such as bacteria, algae, and sponges. Many polyketides have been shown to have activity against a range of bacteria, fungi, and viruses. For example, the marine-derived lindgomycin, an unusual antibiotic polyketide from a marine fungus of Lindgomycetaceae, has been shown to have strong activity against S. aureus and MRSA [40].

Alkaloids are a class of nitrogen-containing compounds produced by marine organisms, including sponges, tunicates, and algae. Many alkaloids have been shown to have antimicrobial activity, making them promising candidates for use as disinfectants. For example, the alkaloid kuanoniamine A from a marine sponge [41] has been shown to have activity against MRSA. Auranomides A and B, quinazolin-4-ones substituted with a pyrrolidin-2-iminium moiety from the marine-derived fungus *Penicillium aurantiogriseum* were found to represent a new scaffold [42]. Unfortunately, many alkaloids have strong cytotoxic properties limiting their use as drugs. However, their use as disinfectants for surfaces might still be an option [43].

Halogenated compounds (from different chemical groups) are frequently isolated from marine resources and display significant potential for antibiotic and disinfectant activity. Many marine organisms, including algae, sponges, and bacteria, produce halogenated compounds as part of their defense against microbial pathogens. These compounds have been shown to have potent antimicrobial activity against a range of bacteria, viruses, and fungi, making them promising candidates for use as disinfectants. For example, the marine-derived halogenated compound pentabromopseudilin, produced by a number of marine bacteria, has been shown to have broad-spectrum activity against Gram-positive and Gram-negative bacteria, including methicillin-resistant *Staphylococcus aureus* (MRSA) and *Pseudomonas aeruginosa* [44]. Another halogenated compound, bromophycolide A, a macrolide isolated from the Fijian red alga *Callophycus serratus*, has been shown to have activity against *S. aureus*, *S. epidermidis,* and *Enterococcus faecalis* [45].

Some secondary metabolites have been shown to have synergistic effects when used in combination with existing antibiotics. For example, a study found that a combination of the marine-derived terpenoid fucosterol from a brown algae and the antibiotic ampicillin had a synergistic effect against *S. aureus* and *P. aeruginosa* [46] and was able to reverse the high-level erythromycin and lincomycin resistance of *Propionibacterium acnes* [47]. This suggests that combining secondary metabolites with existing antibiotics could be a promising approach for the prevention and treatment of SSTIs.

The emergence of multidrug resistance among the latest generation of pathogens suggests that the discovery of new scaffolds should be a priority. Promising approaches to scaffold discovery include mining underexplored microbial niches for natural products, designing screens that avoid the rediscovery of [12] old scaffolds, and repurposing libraries of synthetic molecules for use as antibiotics [48].

The severe drawback of antibiotics will also be valid for any new structure: each application of antibiotics contributes to the further emergence and selection of resistant mutants. To preserve the effectiveness of antibiotics for the future, it is essential to minimize the inappropriate use of antibiotics in animal production and the human healthcare sector, as well as in developing new strategies [49].

## 5. New Strategies

To address the multiresistance problems associated with classical treatment approaches for skin and soft tissue infections, comprehensive strategies are required. These strategies should focus on improving antibiotic stewardship practices, promoting adherence to treatment guidelines, enhancing infection prevention and control measures, implementing surveillance programs, and stopping the spread of multidrug-resistant organisms. Additionally, the development of alternative treatment modalities including the stabilization of a healthy normal flora will be essential to combat multidrug-resistant infections [50]. Marine resources may significantly contribute to such approaches.

Prevention rather than treatment is key in reducing the spread of resistance. Such strategies comprise all kinds of organizational and behavioral approaches, but will also need to involve strategies for disinfections. Disinfection is a critical process that involves the elimination of microorganisms, including bacteria, viruses, and fungi, to prevent the spread of infectious diseases. Traditional disinfectants often have limitations, such as toxicity, environmental persistence, and the emergence of resistant strains. Therefore, there is a growing need for the discovery and development of alternative disinfection strategies. With respect to SSTI, surface disinfection and biofilm inhibition are of special importance, as most of the causative agents are spread by contact or smear infection [51]. Biofilms play a role in the pathogenesis of many SSTIs, and biofilm formation perturbs the efficacy of antimicrobial agents. Therefore, biofilm inhibition and/or eradication is an important consideration in determining the clinical course of treatment [52]. Again, marine natural products offer a vast and largely untapped resource for the identification of novel antimicrobial compounds, which might be applied for disinfectant purposes rather than drug development. As in the marine environment, biofilms are formed on all surfaces [53], and many marine organisms have evolved anti-biofilm strategies to survive. Biofilms are complex microbial communities that adhere to surfaces and are embedded in a self-produced extracellular matrix. Biofilms are notoriously difficult to eradicate and can cause persistent infections and contamination in various environments. Anti-biofilm-defense mechanisms involve the production of bioactive compounds that can inhibit the growth and proliferation of microorganisms but also a number of non-antibiotic but anti-biofilm compounds. Many of them are quorum sensing inhibitors [54]. 

Quorum sensing (QS) is a communication mechanism used by many bacteria to coordinate their behavior as a collective group. It involves the production, release, and detection of small signaling molecules called autoinducers, which allow bacteria to sense the local population density. QS plays a crucial role in the formation and maintenance of biofilms, which are complex microbial communities encased in a self-produced extracellular matrix. Quorum sensing inhibitors (QSIs) are compounds that interfere with the QS signaling process, disrupting the communication and coordination among bacteria. By inhibiting QS, these compounds have the potential to prevent or attenuate biofilm formation, as well as to enhance the susceptibility of existing biofilms to antimicrobial agents. The use of QSIs as anti-biofilm agents offers several advantages. First, targeting the communication mechanism itself rather than specific bacterial targets reduces the likelihood of resistance development. Unlike traditional antimicrobial agents, which exert selective pressure on individual bacteria, QSIs disrupt the collective behavior of the entire bacterial population. Second, QSIs can inhibit the formation of biofilms, preventing the establishment of highly resistant and persistent biofilm phenotypes. Third, QSIs can sensitize existing biofilms to conventional antimicrobials, making them more susceptible to eradication [55]. Many natural and synthetic compounds have been identified as QSIs, including brominated furanones, being produced by a red algae, *Delisea pulchra* [56]. These QSIs have demonstrated the ability to prevent biofilm formation, reduce the production of extracellular matrix components, and increase the susceptibility of biofilms to antibiotics. Despite the progress made in this field, there are challenges to overcome. QS systems are diverse among bacteria, and the efficacy of QSIs can vary depending on the specific bacterial species or strain. Additionally, the development of QSIs with high selectivity and low toxicity is crucial to ensure their safe and effective use [57]. Next to the development of new compounds targeting the pathogens, future strategies will also involve the support of the health of the normal flora as well as strengthening the skin barrier as important preventive measures.

Marine compounds have gained increasing attention for their potential to support the health of normal flora and strengthen the skin barrier. The normal flora comprises a complex ecosystem of microorganisms being controlled by the host and by microbe–microbe interaction [58]. Marine compounds have shown potential in modulating the normal flora by influencing its composition and activity. For example, certain compounds derived from marine microorganisms have exhibited antimicrobial activity against pathogenic bacteria while preserving the growth and viability of beneficial bacteria. By selectively targeting harmful microorganisms, marine compounds can promote a healthy balance within the normal flora. Bromoalterochromides are derived from marine bacteria and have been found to exhibit antimicrobial activity against a wide range of pathogens, including methicillin-resistant *Staphylococcus aureus* (MRSA) and vancomycin-resistant *Enterococcus faecium* (VRE), while sparing beneficial bacteria such as *Lactobacillus acidophilus*. Cytotoxicity might be an issue that needs to be solved prior to further development [59]. Moreover, certain compounds produced by bacteria of the *Pseudoalteromonas* genus have demonstrated antimicrobial activity against pathogenic bacteria, including *Escherichia coli*, *Pseudomonas aeruginosa*, and *Staphylococcus aureus*. These compounds have been shown to selectively target harmful bacteria while preserving the growth of beneficial bacteria, such as *Lactobacillus* spp. [60]. Various marine bacteria, including *Chromobacterium violaceum*, produce the pigment violacein. It exhibits antimicrobial activity against a range of pathogens, including Gram-positive and Gram-negative bacteria, without negatively affecting the growth of beneficial bacteria such as *Lactobacillus acidophilus* [61].

Marine compounds have been investigated for their prebiotic properties. Prebiotics are non-digestible dietary compounds that selectively promote the growth and activity of beneficial microorganisms. Marine-derived polysaccharides, such as fucoidans, laminarins, and alginates, have demonstrated prebiotic effects by acting as substrates for the growth of specific beneficial bacteria. These prebiotic activities contribute to the maintenance and restoration of a healthy normal flora. However, there are no studies available for the prebiotic support of the human skin flora by these compounds [62].

The skin barrier, composed of the stratum corneum and other layers, plays a critical role in preventing excessive water loss, protecting against external pathogens, and maintaining homeostasis. Marine compounds offer potential benefits in strengthening the skin barrier [63]. Certain compounds, such as marine collagen peptides, have been shown to enhance the synthesis and organization of skin structural proteins, including collagen and elastin, promoting the maintenance and integrity of the skin barrier [64]. Wound healing and skin repair will also be an issue where marine compounds may play a role in the future, especially those derived from algal polysaccharides (for review, see: [65,66,67]).

Additionally, marine compounds possess antioxidant and photoprotective properties. Exposure to environmental stressors, such as UV radiation, can induce oxidative stress and damage the skin barrier. Marine-derived antioxidants, such as polyphenols and carotenoids, have shown protective effects against oxidative stress and UV-induced damage, helping to maintain the integrity and functionality of the skin barrier [68]. An application in sun shielding is being discussed. Marine compounds have also been explored for their moisturizing and hydrating effects on the skin. Ingredients derived from seaweed, such as carrageenans and alginates, have shown an ability to retain water and improve skin hydration, thereby supporting the skin barrier function [69].

## 6. The Long Way to Application: Overcoming Bottlenecks

In recent years, advances in technology and research methods have facilitated the exploration and discovery of marine natural products. Various screening strategies, including bioassay-guided fractionation, high-throughput screening, and metagenomic approaches, have been employed to identify and isolate bioactive compounds from marine organisms. Furthermore, advancements in synthetic chemistry and biotechnology allow for the synthesis and modification of marine natural products to improve their activity, selectivity, and stability [70]. Developing innovative methods to collect marine samples can help overcome the bottleneck of limited access to diverse marine microbial communities. Techniques such as metagenomics, which involves analyzing the collective genetic material from environmental samples, can provide a broader range of potential antibiotic-producing organisms [71]. These developments open up new avenues for the development of effective and environmentally friendly disinfectants and biofilm inhibitors. However, enabling sustainable supply is an issue in using marine resources. Marine biotechnology will provide an answer to that, as most of the true producers are microbes, which can be cultivated even on a large scale, avoiding aquaculture or even wild collection [72,73]. Responsible and sustainable production approaches have to be developed and scaled for the respective groups of organisms [74].

Proper drug development will need a supporting political framework for drug development in the field of antibiotics, which has not been the focus of pharmaceutical companies for many years. The step of bridging the innovation gap seems to have started happening recently. After years of industry-wide disinvestment into antibiotic research, a few large companies have started re-investing in this field [37]. However, support by dedicated funding initiatives, regulatory support, and incentives for IP protection and global collaboration will be necessary to drive on health: healthy humans, healthy animals, and a healthy planet [49].

## 7. Conclusions

In conclusion, marine natural products have tremendous potential as a source of novel antibiotics, disinfectants, and biofilm inhibitors. Their ecological role in marine ecosystems serves as a rationale for the discovery and exploration of these compounds. By harnessing the chemical diversity of marine organisms, we can develop innovative solutions to combat microbial infections, improve public health, and mitigate the challenges associated with biofilm-related issues in various industries. Continued research and investment in the field of marine natural products hold great promise for the future of disinfection and biofilm inhibition.

## Data Availability

Not applicable.
